# Adaptation of Aglycosylated Monoclonal Antibodies for Improved Production in *Komagataella phaffii*

**DOI:** 10.1002/bit.28878

**Published:** 2024-11-14

**Authors:** Yuchen Yang, Neil C. Dalvie, Joseph R. Brady, Christopher A. Naranjo, Timothy Lorgeree, Sergio A. Rodriguez‐Aponte, Ryan S. Johnston, Mary K. Tracey, Carmen M. Elenberger, Eric Lee, Mark Tié, Kerry R. Love, J. Christopher Love

**Affiliations:** ^1^ Department of Chemical Engineering Massachusetts Institute of Technology Cambridge Massachusetts USA; ^2^ The Koch Institute for Integrative Cancer Research Massachusetts Institute of Technology Cambridge Massachusetts USA; ^3^ Department of Biological Engineering Massachusetts Institute of Technology Cambridge Massachusetts USA; ^4^ Biogen Cambridge Massachusetts USA

**Keywords:** biomanufacturing, biopharmaceuticals, fermentation, *Pichia pastoris*, yeast

## Abstract

Monoclonal antibodies (mAbs) are a major class of biopharmaceuticals manufactured by well‐established processes using Chinese Hamster Ovary (CHO) cells. Next‐generation biomanufacturing using alternative hosts like *Komagataella phaffii* could improve the accessibility of these medicines, address broad societal goals for sustainability, and offer financial advantages for accelerated development of new products. Antibodies produced by *K. phaffii*, however, may manifest unique molecular quality attributes, like host‐dependent, product‐related variants, that could raise potential concerns for clinical use. We demonstrate here conservative modifications to the amino acid sequence of aglycosylated antibodies based on the human IgG1 isotype that minimize product‐related variations when secreted by *K. phaffii*. A combination of 2–3 changes of amino acids reduced variations across six different aglycosylated versions of commercial mAbs. Expression of a modified sequence of NIST mAb in both *K. phaffii* and CHO cells showed comparable biophysical properties and molecular variations. These results suggest a path toward the production of high‐quality mAbs that could be expressed interchangeably by either yeast or mammalian cells. Improving molecular designs of proteins to enable a range of manufacturing strategies for well‐characterized biopharmaceuticals could accelerate global accessibility and innovations.

## 1 Introduction

Recombinant monoclonal antibodies (mAbs) are one of the most important classes of biopharmaceuticals and one of the fastest growing classes of biologic medicines by the number of newly approved products and biosimilars, numbers of patients treated, and total revenue (Ecker, Jones, and Levine 2015). Advances in single‐cell screening and machine learning algorithms to predict binding and manufacturability have accelerated the design, discovery, and optimization of mAbs as therapeutics in the past 10 years (Makowski et al. 2022; Williams et al. 2024). Manufacturing these medicines presently relies on highly standardized processes primarily using Chinese hamster ovary (CHO) cells for recombinant expression and protein A (ProA)‐based chromatography for recovery, with new advances in continuous manufacturing emerging for clinical and commercial use (Coffman et al. 2021).

There remain both societal and commercial considerations that necessitate the evolution of strategies for next‐generation biomanufacturing of these drugs and related ones (like bi‐specific antibodies and single‐chain antibodies). Some factors include improving the accessibility of these medicines for global populations (Kelley, Renshaw, and Kamarck 2021), increasing requirements for sustainable bioprocessing (Lalor et al. 2019), reducing time to market as a key business advantage, and responding rapidly to global events like pandemics (Brady and Love 2021). For example, the high cost of goods manufactured (COGSm), which today can range from $30–100 per gram of antibody (Shukla and Thömmes 2010), is one of two major constraints to access of these medicines in low‐ and middle‐income countries (LMICs); indeed, 80% of all doses for registered products are administered only in North America and Europe (IAVI/Wellcome 2020). Corporate commitments to reduce carbon footprints and geopolitical interests for enabling a circular bioeconomy also underscore the importance for continued developments to intensify, consolidate and reduce energy, water, and raw materials used in bioprocessing (Bunnak et al. 2016; Pietrzykowski et al. 2013). Finally, accelerated development demonstrated during the COVID‐19 pandemic showed the potential to reduce the time from discovery to first‐in‐human clinical trials to 4–6 months, albeit with significant corporate and regulatory cooperation (Kelley, 2020). These examples emphasize the possibility for more rapid development, clinical assessment, and commercialization of innovative new biopharmaceuticals. Together, these factors motivate the need for continued innovations in the approaches to manufacture mAbs (and other recombinant proteins) with shorter development timelines, lower production costs, and improved sustainability.

The existing infrastructure to manufacture therapeutic mAbs has evolved through the continuous improvement of now standardized “platform” processes using CHO cells (Kelley, Kiss, and Laird 2018). The space‐time yields for state‐of‐the‐art fed‐batch processes can reach ~0.2–0.5 g/L/day with fully continuous processing achieving up to ~2–4 g/L/day (Kelley, Kiss, and Laird 2018; Mahal, Branton, and Farid 2021). With these outputs, the costs of drug substance could approach ~$30 per gram, albeit with limited additional gains expected without new technologies for recovery (Kelley, Renshaw, and Kamarck 2021; Mahal, Branton, and Farid 2021). Ultimately, reducing COGSm (and improving sustainability of bioprocessing) requires maximizing space‐time yields from the smallest facilities with reduced labor (Pollock et al. 2017). Additional gains will result from removing process operations, increasing automation, and reducing biological variations through improved control or host biology (Crowell et al. 2018, 2021).

The biology of unconventional or alternative hosts can directly increase the volumetric output of production through faster doubling times and reduced process requirements (Brady and Love 2021; Coleman 2020; Jiang et al. 2019). The yeast *Komagataella phaffii* (*Pichia pastoris*) is particularly well‐suited for producing mAbs because it contains an advanced secretory pathway with a stacked Golgi apparatus, secretes few native host cell proteins, contains process‐inducible genetic promoters, and is Generally Recognized As Safe (GRAS) by global regulators (Love, Dalvie, and Love 2018). Indeed, *K. phaffii* is routinely used to manufacture therapeutic proteins like insulin and subunit vaccines both in both high‐income countries and LMICs (Shekhar 2008). Producing mAbs with *K. phaffii* has been considered (Dodick et al. 2014; Li et al. 2006; Potgieter et al. 2009), but faces certain challenges from the intrinsic biology. Yeast‐like glycosylation imparted on expressed proteins may not be suitable for many products, though this variation can be modified by strain engineering (Choi et al. 2003; Hamilton et al. 2006; Jacobs et al. 2009; Vervecken et al. 2004). Proteolysis of the target sequences can also impact yield and quality for this yeast and other microorganisms (Gil et al. 2011; Kerry‐Williams et al. 1998; Sinha et al. 2005).

Here, we have considered an alternative approach to advance the utility of *K. phaffii* for producing high‐quality, full‐length aglycosylated human immunoglobulin IgG1. Aglycosylated antibodies have emerged as an important new engineered subclass for these drugs (Jung et al. 2011). Advances in protein engineering make it possible to modulate the engagement of Fc receptors to minimize immunological effector functions in vivo (Ju and Jung 2014). In 2020, the FDA‐approved eptinezumab (Vyepti), an aglycosylated mAb manufactured by Alder/Lundbeck in *K. phaffii* for treatment of chronic migraines (Dhillon 2020). Several other aglycosylated mAbs have been approved or are in clinical trials for indications ranging from diabetes to non‐small‐cell lung cancer (NSCLC) (Mimura et al. 2017). Given this emerging use of engineered sequences, we sought here to adapt the sequence of the common human IgG1 mAb to reduce the typical variations observed in yeast‐expressed mAbs. We postulated that conservative strategies for protein engineering (similar to those that we have applied previously to subunit vaccine candidates (Dalvie, Brady, et al. 2021; Dalvie, Rodriguez‐Aponte, et al. 2021; Rodriguez‐Aponte et al. 2023) could improve the quality attributes while introducing minimal or no adverse effects on protein function. Specifically, we present here studies on the vector designs, including the signal peptides, and the sequence‐related liabilities in the human IgG1 mAb that affect the proteolysis of the heterodimer. We show that minimal variations (2–3 amino acids) can improve the quality attributes of seven aglycosylated variants of mAbs in *K. phaffii*. Finally, we demonstrate that the quality of the engineered mAb secreted by *K. phaffii* is comparable to that produced by industrial‐grade CHO cells.

## 2 Materials and Methods

### 2.1 Yeast Vectors and Strains

The custom vector was constructed by synthesis of DNA fragments (IDT) and Gibson assembly (New England Biolabs). Genes containing mAb light and heavy chains were codon optimized, synthesized (Integrated DNA Technologies), and cloned into the custom vector. Modification of the vector, including replacement of signal peptides and addition of mutations to the IgG1 sequence were performed using PCR and site‐directed mutagenesis (NEB). All yeast strains were derived from wild‐type *K. phaffii* (NRRL Y‐11430). After initial screening of signal peptides, all strains were derived from a modified base strain (AltHost Research Consortium Strain S‐63 [RCR2_D196E, RVB1_K8E]) described previously (Brady et al. 2020). *K. phaffii* strains were transformed as described previously (Dalvie et al. 2019).

### 2.2 Yeast Cultivations

Strains for initial characterization and titer measurement were grown in 3 mL culture in 24‐well deep well plates (25°C, 600 rpm), and strains for protein purification were grown in 100 mL culture in 500 mL shake flasks (25°C, 300 rpm). Cells were cultivated in complex media (potassium phosphate buffer pH 6.5, 1.34% nitrogen base w/o amino acids, 1% yeast extract, 2% peptone). Cells were inoculated at 0.1 OD600, outgrown for 24 h with 4% glycerol feed, pelleted, and resuspended in fresh media with 1% methanol and 40 g/L sorbitol to induce recombinant gene expression. Supernatant samples were collected after 24 h of production, filtered, and analyzed.

### 2.3 CHO Vector and Strains

The heavy and light chain coding sequences for NISTmAb (with or without the described mutations) were codon optimized (Raab et al. 2010) for expression in *C. griseus* and inserted via restriction digestion into two otherwise identical expression plasmids that differ only in their metabolic selection markers. Each respective pair of expression plasmids was cotransfected into CHO host cells, previously described elsewhere (Wright et al. 2017). Selective pressure was applied 24‐h posttransfection through a complete media exchange into chemically defined medium lacking critical metabolites. Transfectant pools were cultured at 36°C and 5% CO_2_ and resuspended into fresh selection medium every 3–7 days until the cell pools returned above 90% viability.

### 2.4 CHO Fed‐Batch Material Generation

CHO‐derived NISTmab was produced in a 14‐day fed‐batch process. Recovered transfectant pools were seeded at a target density of 1 × 10^6^ cells/mL into a proprietary chemically defined production medium (Huang et al. 2010) with an initial working volume of 100 mL in a 500 mL flask. Fed‐batch cultures were cultivated under shaking conditions at 5% CO_2_ and 35°C until Day 7, when the temperature was lowered to 31°C. Beginning on Day 2, daily fixed volumes of a proprietary chemically defined complex feed were added to sustain the culture duration. On Day 14, cell culture supernatants were harvested by centrifugation and filtration before final storage at −70°C.

### 2.5 Protein Purification

The filtered supernatant samples were diluted, 1:1 with 1x phosphate‐buffered saline (PBS, pH 7.4) for 3‐mL culture or 10:1 with 10x PBS for 100‐mL culture, and purified on the GE ÄKTA pure system with a 1‐mL ProA column (HiTrap Protein A HP, Cytiva). The column was equilibrated with 10 CVs of 1xPBS at a rate of 1.0 mL/min. After sample loading, the column was washed with 1xPBS for 5 CVs at 1.0 mL/min, and eluted with 100 mM citric acid (pH 2.8) for 7 CVs at 1.0 mL/min. Elution above 6 mAu was collected. Eluted products were pH adjusted with 1M Tris‐HCl (pH 9.0) and stored at 4°C.

### 2.6 Exoglycosidase Digestion

Approximately 30–50 μg of protein was digested with PNGaseF or α1‐2,3,6‐mannosidase (New England Biolabs) according to manufacturer's recommended protocol. After overnight incubation, the reaction mixture was analyzed using SDS‐PAGE. For downstream mass spectrometry analysis, acetone precipitation was carried out as preparation. Briefly, 4x volume of cold acetone was added to the reaction mixture. Samples were then gently vortexed and incubated at −20°C for 1 h. Proteins were then precipitated by centrifugation at 15,000 g for 15 min at −10°C. The supernatant was decanted, and the air–dried protein pellets were resuspended in 5% acetone in water with 0.1% formic acid.

### 2.7 Analytical Assays for Protein Characterization

Purified protein concentrations were determined by absorbance at A280 nm. SDS‐PAGE was carried out under reducing conditions using Novex 12% Tris‐Glycin Midi Gels (Thermo Fisher Scientific). Separation was performed at 115 V for 100 min. Gels were stained with Instant Blue Protein Stain (Abcam Inc) and destained with deionized water for a total of three times before imaging.

Biolayer interferometry was performed using the Octet Red96 with ProA biosensors (Sartorius ForteBio), which were hydrated for 15 min in kinetics buffer (1x PBS, 0.5% BSA, and 0.05% Tween 20) before each run. Kinetics buffer was used for all dilutions, baseline, and dissociation steps. Seven 1:1 serial dilutions of trastuzumab standard and one 1:1 dilution of each sample were loaded in a 96‐well black microplate (Greiner Bio‐One). Association and dissociation were measured at 1000 rpm for 300 and 600 s, respectively. Binding affinity (and sample concentration) were calculated using the Octet Data Analysis software v10.0 (Pall ForteBio), using reference subtraction, baseline alignment, inter‐step correction, Savitzky‐Golay filtering, and a global 1:1 binding model. Specific productivity was defined as relative titer normalized by cell density, measured by OD600.

Size exclusion chromatography, intact mass spectrometry, and far‐ultraviolet circular dichroism were performed as described previously (Dalvie, Brady, et al. 2021).

## 3 Results

We aimed to define a generalizable strategy to express high‐quality, aglycosylated mAbs in *K. phaffii*. Toward this goal, we first analyzed the common molecular variations manifest in a monoclonal antibody expressed by *K. phaffii*. We assessed the impact of the expression vector and recombinant protein sequence on mAb expression in *K. phaffii* for a commonly studied IgG1κ mAb—trastuzumab (trade name Herceptin). This molecule is well characterized and has been previously expressed in *K. phaffii* (Liu et al. 2011; Zhang et al. 2011). It is also a useful model since most approved mAbs (and in clinical development) contain the IgG1κ constant region.

We designed a new vector to allow for rapid cloning and testing in *K. phaffii*. Commercial vectors are available to express recombinant proteins in *K. phaffii* at lab and industrial scales, but these predominantly rely on traditional methods for molecular cloning such as enzyme‐mediated restriction digestion (User Manual 2010). Our designed plasmid contains a promoter‐gene‐terminator cassette for expression of a transgene, and a second cassette for expression of a selection marker. Each vector element is separated by eight unique 40 base pair DNA linker sequences, which enable rapid swapping of individual vector parts by Gibson assembly. This design allowed us to interchange different promoters, signal sequences, and gene sequences of interest here.

We used this vector to create expression cassettes for both the heavy and light chains of trastuzumab and integrate them into a modified strain of *K. phaffii* (AltHost S‐63) to evaluate the expression of each component. We expressed the heavy chain using the canonical methanol‐inducible P_AOX1_ as the promoter and integration locus. We also expressed the light chain using another previously identified strong, methanol‐inducible promoter, P_DAS2_, as the promoter and integration locus (Love et al. 2016). We constructed expression vectors for both light chain and heavy chain using the described custom vectors. Previous reports have shown that a hybrid secretion signal comprising the preregion of *Saccharomyces cerevisiae* OST1 signal sequence and the pro region of *α*‐mating factor signal peptide (αSP) can significantly improve recombinant protein expression in *K. phaffii* by promoting co‐translational translocation (Barrero et al. 2018). We elected to use this preOST1‐proαSP in our initial studies without the Glu‐Ala‐Glu‐Ala (EAEA) sequence found at the end of the native αSP. The Golgi‐resident dipeptidyl aminopeptidase STE13 can remove this EAEA sequence (Lin‐Cereghino et al. 2013), but the efficiency of cleavage varies by molecule (leading to *N*‐terminal variations on the recombinant protein) (Dalvie, Brady, et al. 2021; Kozlov and Yagudin 2008). Since the processing of αSP can occur without the EAEA motif (Ghosalkar, Sahai, and Srivastava 2008), the four‐amino acid sequence is often omitted (Neiers et al. 2021; Wang et al. 2012). This dual expression system allowed us to test variations in both overall and relative copy number (Supporting Information S1: Figure S1).

We analyzed the variations manifest in trastuzumab when secreted from *K. phaffii* following cultivation in flasks and purification by ProA chromatography. Product‐related variations in the drug substance of a biopharmaceutical can reduce productivity of the cell culture, increase the complexity and costs for purification, and may introduce risks for patients (Gramer 2014). Variations of mAbs expressed by CHO cells can include *N*‐terminal variations, modulated glycosylation, sulfation, phosphorylation, hydroxylation, carboxylation, amidation, glycation, and misfolding, among others (Beck and Liu 2019; Walsh and Jefferis 2006). Aside from those variants resulting from native yeast glycans, detailed assessments of other variations of mAbs secreted from *K. phaffii* have not been well‐documented.

**Figure 1:**
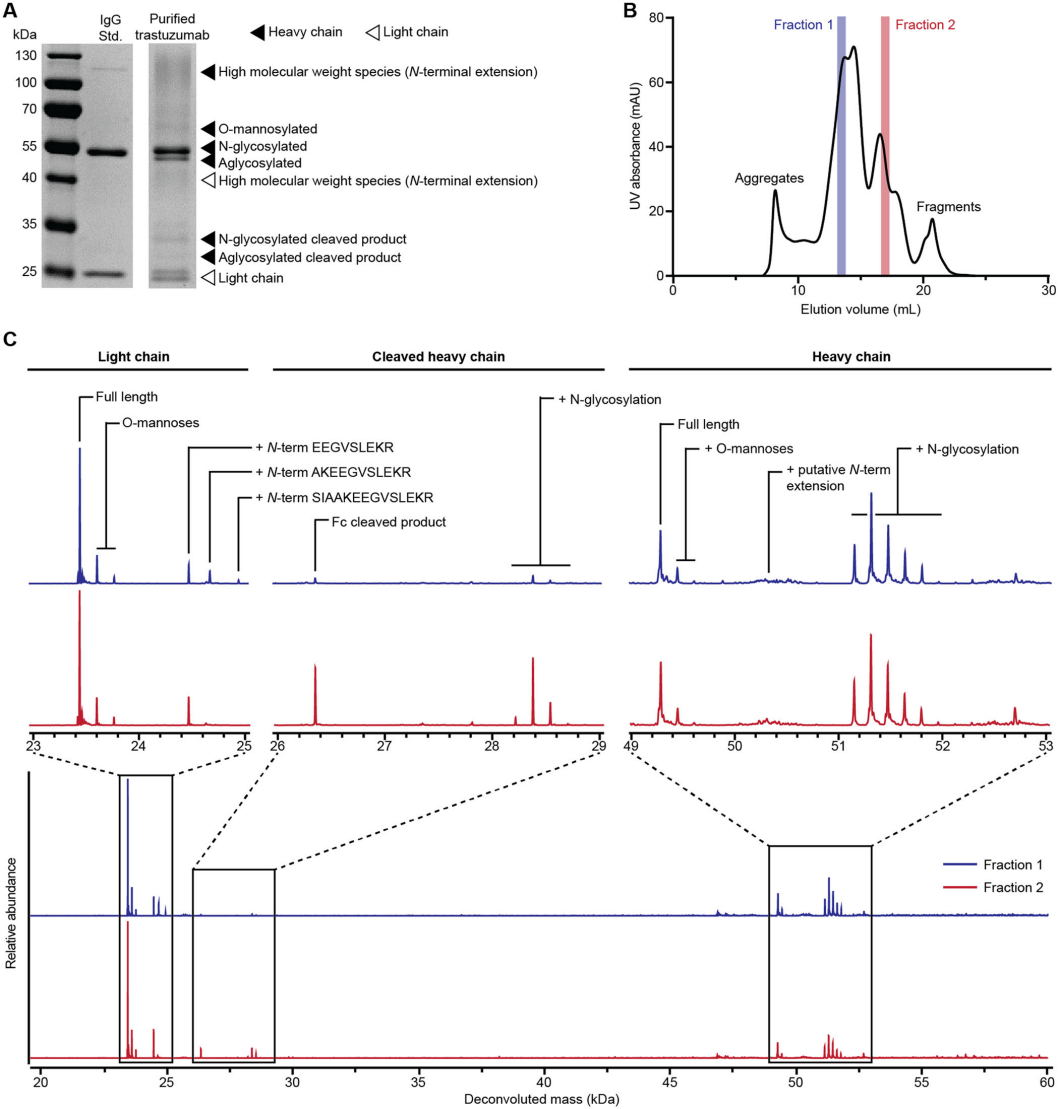
Characterization of product‐related variants of *Komagataella phaffii*‐secreted trastuzumab. (A) Reduced SDS‐PAGE of protein A‐purified trastuzumab secreted from *K. phaffii*. (B) Analysis of purified trastuzumab sample by size exclusion chromatography, with two fractionations of interest highlighted in red and blue. (C) Mass spectra of the two fractionations analyzed by intact protein LC‐MS, with zoomed‐in views of light chain, heavy chain, and heavy chain Fc cleavage product.

The purified trastuzumab showed many product‐related variants (Figure 1A). To differentiate these variants, we fractionated the purified protein with size exclusion chromatography (Figure 1B) and evaluated the predominant fractions by intact mass spectrometry (Figure 1C). There were three major product‐related variants. First, we observed high molecular weight (HMW) variants of both the trastuzumab heavy chain and light chain. Observed mass shifts for the HMW variants of light chain were consistent with *N*‐terminal extensions resulting from incomplete cleavage of the proαSP (Supporting Information S1: Figure S2). The identities of the HMW species of heavy chain were more difficult to determine, likely due to the presence of glycans. To facilitate our analysis, we treated the purified mAb with PNGase to cleave N‐linked glycans and confirmed the presence of similar *N*‐terminal extensions due to incomplete cleavage of the proαSP on the heavy chain (Supporting Information S1: Figure S3). This portion of the proαSP should be removed in the Golgi apparatus of *K. phaffii* by cleavage at a dibasic motif KR by KEX2 protease (Lin‐Cereghino et al. 2013). The proαSP contains both N‐ and O‐linked glycosylation sites and these modifications may also contribute to the formation of HMW species when the *N*‐terminal extension is not efficiently removed. This observation is similar to ones we have reported previously for subunit protein vaccine antigens secreted from *K. phaffii* (Dalvie, Brady, et al. 2021).

Second, we observed a variant with a slightly higher apparent mass than the unmodified heavy chain by SDS‐PAGE. Treatment with PNGase did not remove this variant, but incremental mass shifts of 161 Da are consistent with hexose addition. Furthermore, digestion with α1‐2,3,6 mannosidase removed this variant, suggesting that the product variant may contain *O*‐glycosylation (Supporting Information S1: Figure S4). We attempted to identify potential sites of *O*‐glycosylation on this variant by performing an in‐gel tryptic digest and LC‐MS/MS. We found only one tryptic peptide [STSGGTAALGCLVK] early in the IgG1 heavy chain constant region that appeared differentially *O*‐glycosylated compared to the unmodified heavy chain (Supporting Information S1: Figure S5). We tested several sequence variants that removed the Ser and Thr residues in this peptide, but we still observed *O*‐glycosylated variants by SDS‐PAGE (Supporting Information S1: Figure S6), suggesting there may be other potential sites for *O*‐linked mannosylation. The observed degree of *O*‐mannosylation is low, and similar posttranslational modification has been reported in mAbs produced by higher eukaryotes including CHO and COS cells as single‐mannose additions on the light chain of IgG2 (Martinez et al. 2007).

Third, we observed a cleaved heavy chain fragment. We performed intact LCMS and identified the variant as a *C*‐terminal fragment of the trastuzumab heavy chain (Supporting Information S1: Figure S7). Interestingly, this fragment begins near a dibasic motif (K/KVE…) that may also be a cleavage target for KEX2 protease. While KEX2 cleavage canonically occurs at the *C*‐terminal side of a dibasic motif, additional mass adducts or losses may confound the intact mass analysis.

**Figure 2:**
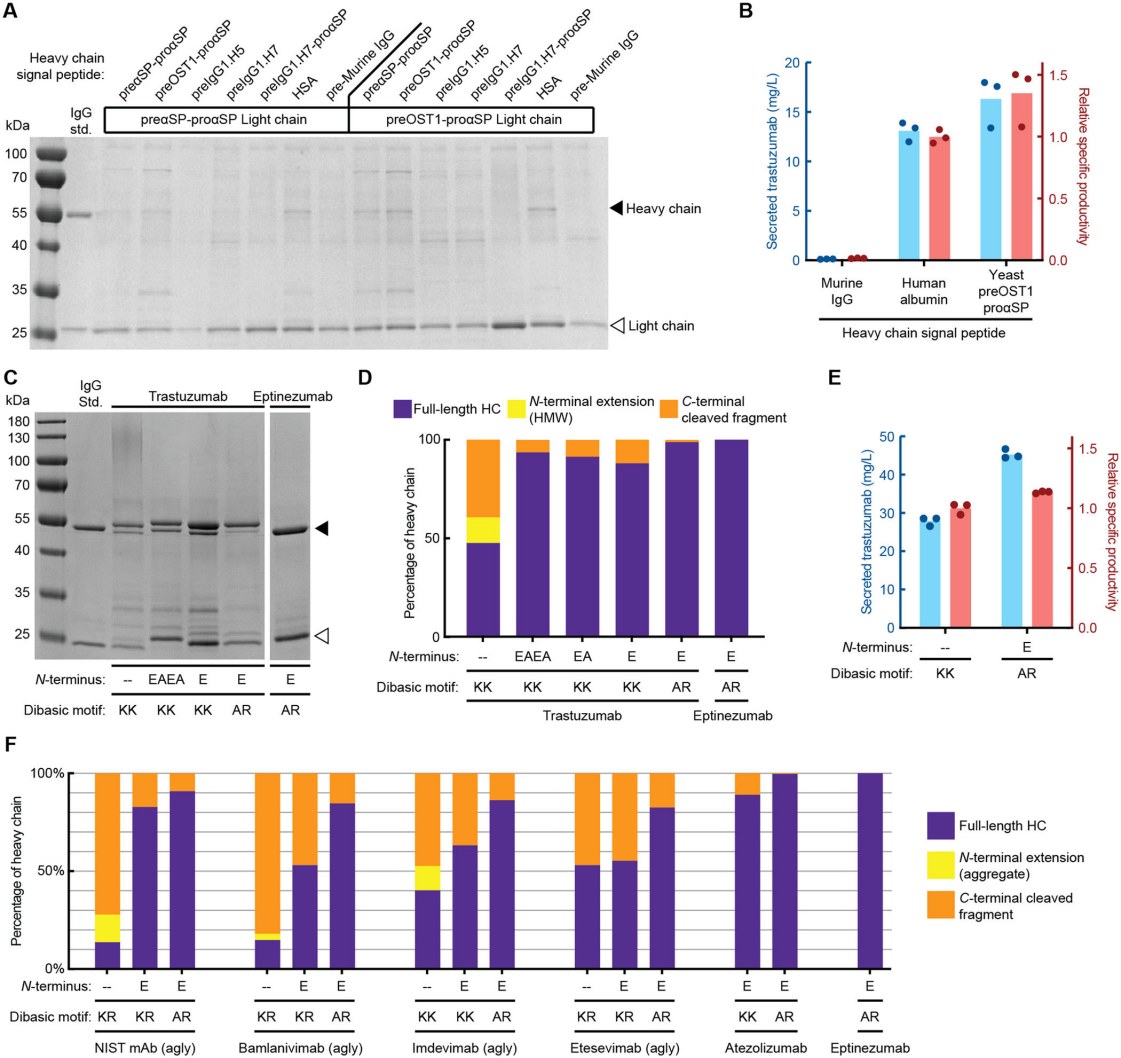
Engineering of IgG1 sequence to eliminate product‐related variants. (A) Reduced SDS‐PAGE of culture supernatants from *Komagataella phaffii* producing trastuzumab with different signal peptides. (B) Secreted titer and specific productivity of trastuzumab from a wild‐type *K. phaffii* strain with different heavy chain signal peptides. All strains use the preOST1‐ proαSP for the light chain. (C) Reduced SDS‐PAGE of mAb variants secreted from *K. phaffii* and purified by protein A chromatography. (D) Abundance of heavy chain variants, determined by intact LC‐MS of purified mAb variants. Abundance was normalized within samples. (E) Secreted titer and specific productivity of trastuzumab variants from the modified *K. phaffii* base strain. (F) Abundance of heavy chain variants, determined by intact LC‐MS of purified variants of six mAbs. Abundance was normalized within samples.

Given our observations on incomplete cleavage of the signal peptide from the expressed heavy and light chains, we decided to test other signal peptides, including ones that direct native secretion of immunoglobins in other organisms, to assess how these could alter the *N*‐terminal extensions observed. The signal peptide, which directs translocation of the polypeptide into the endoplasmic reticulum, can impact the both secreted titers and quality, likely due to the difference in timing between translation and translocation and in subsequent processing in the yeast secretory pathway (Hegde and Bernstein 2006). The most commonly used signal peptide for expression of recombinant proteins in *K. phaffii* is the signal peptide from the *S. cerevisiae* alpha mating factor gene (αSP) (Lin‐Cereghino et al. 2013). Previous reports of mAb expression in *K. phaffii* have suggested that alternative signal peptides from the *K. phaffii* genome or from other eukaryotic organisms may yield higher secreted titers of mAbs (Aw et al. 2018). We first evaluated the expression of the trastuzumab light chain with seven additional signal peptides, and observed the highest secreted titers with αSP or the preOST1‐proαSP. We then tested the expression of the trastuzumab heavy chain vectors with different signal peptides in strains containing expression cassettes for the light chain bearing these two signal peptides (αSP or preOST1‐proαSP). Overall, we observed higher secreted titers of both chains when the light chain was expressed with the preOST1‐proαSP (Figure 2A). The αSP, preOST1‐proαSP and the signal peptide from human serum albumin (HSA‐SP) yielded the highest secreted titers of the trastuzumab heavy chain (as measured by ProA biolayer interferometry). These combinations yielded a 79‐fold higher specific productivity compared to the murine immunoglobin signal peptide preregion previously reported for expressing mAbs in *K. phaffii* (Figure 2B) (Aw et al. 2018). There was no significant improvement in product quality evident by SDS‐PAGE, though the titers for many of tested signal peptides were likely not high enough to observe the changes in product‐related variants. These data together suggested the best constructs from this set of peptides tested still used the preOST1‐proαSP signal sequence, and we chose to use this sequence for further engineering to improve product quality.

We then examined the copy number ratio between the light chain and heavy chain vectors to explore its impact on the secretion of the mAb. It is typical to evaluate the secreted expression of several *K. phaffii* transformants to identify an optimal clone for production of a heterologous recombinant protein. In theory, transformants differ primarily by the copy number of the vector integrated into the host genome. Higher copy numbers can yield higher levels of the recombinant transcript, and in turn improve the secreted titer of simple recombinant proteins (Yang et al. 2016). High transcript levels of complex proteins, however, may activate other competitive pathways such as endoplasmic reticulum‐associated degradation (ERAD) and the unfolded protein response (UPR) (Delic et al. 2014). Lower copy numbers may be beneficial, therefore, for more complex proteins. Previous studies have suggested that the light chain can properly fold and secrete without the heavy chain, but that the heavy chain has more complex folding requirements and requires the light chain for efficient folding and secretion (Haryadi et al. 2015).

We transformed a vector encoding the heavy chain of trastuzumab modified to remove the site for N‐linked glycosylation (N300A) into a base strain of *K. phaffii*. We selected three transformants that exhibited high, medium, or low growth on selection plates, as an approximation for high, medium, and low copy numbers of the heavy chain vector. We then transformed a vector encoding the trastuzumab light chain into each of these transformants and identified 6‐8 transformants from each that exhibited a range of growth rates on selections plates, as an approximation for the copy number of the light chain vector. We evaluated the secreted expression of all transformants and observed a positive correlation between secretion of the light chain and secretion of the heavy chain (Supporting Information S1: Figure S8). To test if this correlation was universal or specific to trastuzumab, we performed the same series of transformations with a second human IgG1 mAb that is currently in preclinical development (mAb1). Interestingly, we observed a negative correlation between secretion of the mAb1 light chain and the mAb1 heavy chain. These results suggest that the optimal ratio of light chain copy number to heavy chain copy number depends on the nature of the recombinant mAb and the *K. phaffii* strain background. Although different copy number ratio between light chain and heavy chain yielded different titers, we did not observe any correlation between copy number ratio and product‐related variants, based on SDS‐PAGE.

We then examined if product‐related variants of trastuzumab could be reduced by modifying the sequence. We and others have shown that small, conservative changes to the amino acid sequence of therapeutic proteins can have large impacts on quality and manufacturability (Dalvie, Brady, et al. 2021; Dalvie, Rodriguez‐Aponte, et al. 2021). Sequence engineering has also improved the quality and manufacturability of mAbs (Pettit et al. 2016). We previously reported that addition of amino acid residues to the *N*‐terminus of a recombinant protein can reduce *N*‐terminal extensions, likely by increasing the steric accessibility of the KEX2 cleavage site (Dalvie et al. 2022). We hypothesized that this strategy may also reduce *N*‐terminal extension of the light chain and heavy chain of trastuzumab. We expressed and purified trastuzumab with EAEA, EA, or E residues added to the *N*‐terminus of both the heavy chain and light chain (Figure 2C). We observed nearly complete elimination of *N*‐terminal extension with EAEA addition, and significant reduction thereof with the addition of a singular E residue. Interestingly, we also observed reduction of the *O*‐glycosylated heavy chain variant and the cleaved *C*‐terminal heavy chain fragment. We analyzed each purified sample by intact LCMS and observed that residues attached to the *N*‐terminus of the heavy chain and light chain remained on the purified protein (Supporting Information S1: Figure S9). This observation is consistent with our previous observations for several subunit vaccine antigens (Dalvie et al. 2022).

Next, we sought to further reduce or eliminate the *C*‐terminal heavy chain fragment. We analyzed the sequence of the recently approved mAb for headache treatment, eptinezumab (Vyepti), that is manufactured in *K. phaffii* (Dhillon 2020). Eptinezumab has two sequence modifications (K207A, K208R) that removed the dibasic motif in the heavy chain constant region that may be the cause of *C*‐terminal cleavage product observed for trastuzumab. We expressed eptinezumab in *K. phaffii* using preOST1‐proαSP with a single E residue on the *N*‐terminus of both the light chain and heavy chain and purified the modified eptinezumab by ProA chromatography. We did not observe any *C*‐terminal heavy chain fragment (Figure 2C).

To evaluate if this change could confer the same benefit to trastuzumab, we created a variant with the same mutations. We quantified the relative abundance of the two major heavy chain variants by LCMS and observed a large reduction in the abundance of the *C*‐terminal heavy chain fragment—the detected fraction of cleaved heavy chain dropped from ~11% to < 1% with K207A and K208R mutations (Figure 2D). We also evaluated the secreted titer of trastuzumab with and without both sequence modifications and observed that the modified trastuzumab molecule was secreted with slightly increased specific productivity (Figure 2E). The strain secreting the modified trastuzumab also grew to a higher cell density, which resulted in a ~60% increase in secreted titer. This result suggests that reduction or elimination of product‐related variants also improved cellular processing and secretion of the mAb, likely because misfolded or incorrectly modified mAb molecules may trigger degradation pathways or stress responses in the yeast secretory pathway, reducing the overall secreted titer (Delic et al. 2013).

We then tested the benefits of these changes for five additional IgG1 sequences, including NIST mAb, bamlanivimab, imdevimab, etesevimab, and atezolizumab. We mutated the conserved *N*‐linked glycosylation site with N‐to‐A mutation in the heavy chain Fc region in NIST mAb, bamlanivimab, imdevimab, and etesevimab to reduce product quality variability due to glycosylation. Analysis with LCMS showed improvement in the fraction of full‐length heavy chain in all mAbs tested (Figure 2F). Overall, these results demonstrate that small modifications to the human IgG1 backbone can greatly improve both the quality and titer of mAbs secreted from *K. phaffii*.

**Figure 3:**
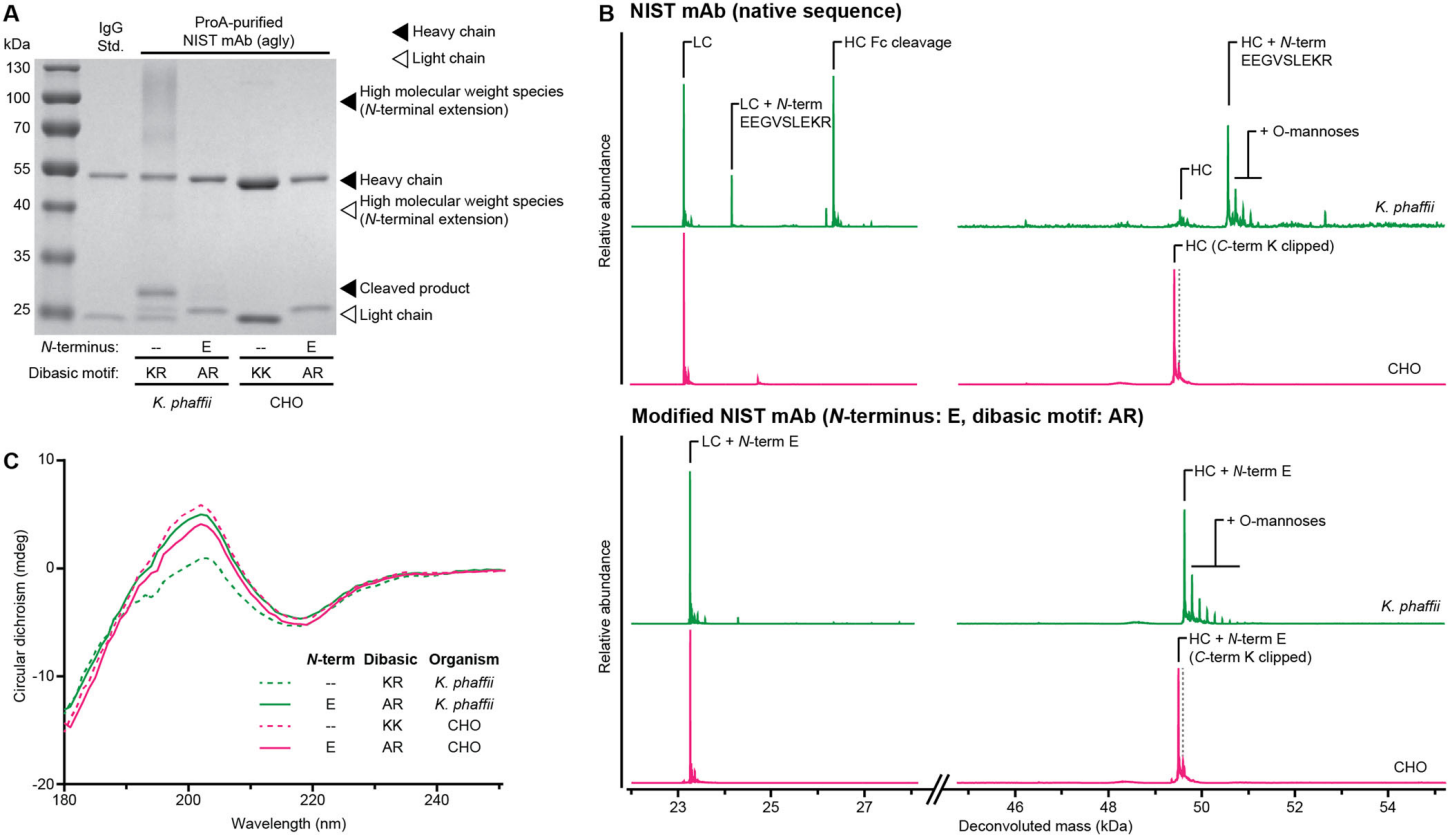
Product quality comparison of mAb variants secreted from *Komagataella phaffii* and from CHO. Reduced SDS‐PAGE (A), intact LC‐MS mass spectra (B), and ultraviolet circular dichroism spectra (C) of aglycosylated NIST mAb variants secreted from *K. phaffii* and from CHO and purified by protein A chromatography.

One potential use for yeast‐based expression of aglycosylated mAbs would be to support rapid production for first‐in‐human clinical studies (Brady and Love 2021). It is feasible to consider changing the host used for later stage clinical trials and commercialization of such mAbs following demonstration of analytical and, if necessary, clinical comparability (Ratih et al. 2021). To evaluate if these modifications to IgG1 backbone are transferrable to other expression systems, we compared the secreted aglycosylated NIST mAb with and without sequence engineering to those secreted by CHO. We saw a significant amount of N‐terminal extension and heavy chain Fc cleavage in the unmodified yeast‐secreted product by SDS‐PAGE (Figure 3A). *N*‐terminal E‐addition and the dibasic site mutation essentially eliminated these product‐related variants. Aglycosylated NIST mAbs secreted from CHO, both unmodified and engineered, showed no *N*‐terminal extension or heavy chain cleavage, but they did exhibit *C*‐terminal lysine clipping, a known posttranslational modification by endogenous carboxypeptidases during CHO cultivation (Figure 3B) (Brorson and Jia 2014). We then compared the secondary structures of both the yeast‐ and CHO‐secreted products. Far‐ultraviolet circular dichroism spectroscopy showed converging confirmational profiles among both CHO‐secreted molecules and the engineered yeast‐secreted product. Purified un‐engineered yeast product showed a different CD profile, likely due to *N*‐terminal extension and heavy chain cleavage (Figure 3C). These data show that the modified IgG1 sequence can be expressed in both *K. phaffii* and CHO cells with highly similar structures and minimal differences.

## 4 Discussion

In this study, we developed a modular approach to express aglycosylated antibodies in *K. phaffii*. By using a dual integration system with our customized vectors, we secreted full‐length trastuzumab, but several apparent product‐related variants were evident with the original sequence. After characterizing the variants and identifying them as an *N*‐terminal extension and heavy chain Fc cleavage, we modified the amino acid sequence for the human IgG1 in two places to eliminate most product‐related variants after affinity purification with ProA. These sequence changes resulted in higher titers of trastuzumab in flasks, likely due to the reduction of variants. These engineering changes also improved the quality of five other aglycosylated variants of biopharmaceutical IgG1's when expressed in *K. phaffii*, greatly reducing N‐terminal signal peptide extension and Fc cleavage.

These conservative modifications have prior clinical precedents that reduce their potential risks for clinical or commercial use. The modification of the dibasic site motif has been used in Vyepti (eptinezumab). Across IgG subclasses, this motif presents as KK in IgG1, KR in IgG3 and IgG4, and KT in IgG2 (Alan Lazar and Llp 2007). The clinical precedent and the intrinsic variability demonstrate tolerance for mutations at this motif. The beneficial addition of a single *N*‐terminal amino acid is similar to the intrinsic modification found on recombinant proteins produced by bacteria such as *E. coli*. Due to the absence of a secretory pathway (and thus no secretion signal peptide processing) in prokaryotic hosts, an extra *N*‐terminal methionine (which initiates recombinant protein translation) is present in the final drug product (Xiao et al. 2010). Myalept (met‐leptin), Neupogen (met‐G‐CSF), and Protropin (met‐hGH, with second generation product Nutropin, hGH) are among commercial recombinant methionyl pharmaceutical proteins (Walsh and Walsh 2022). The *N*‐terminal methionine from bacteria‐produced proteins can be cleaved by peptidases, but residual variants with single amino acid *N*‐terminal extensions have been present in biologic medicines. We have previously shown that the identity of this single amino acid can also vary—including methionine—to give similar reductions of *N*‐terminal variations resulting from incomplete signal peptide cleavage (Dalvie et al. 2022).

With these combined conservative changes, we showed the quality of secreted aglycosylated mAb products were highly comparable between yeast and CHO, the industry standard. In fact, yeast‐secreted NIST mAb did not exhibit any C‐terminal lysine clipping, a common product‐related variant in CHO cultures (Dick et al. 2008). The engineered, aglycosylated NIST mAb showed similar secondary structures as the unmodified NIST mAb expressed by CHO, indicating that these minimal sequence changes did not significantly alter the overall protein conformation. The *K. phaffii*‐secreted product did exhibit minor *O*‐linked mannosylation. Similar mannosylated variants have been reported to be present in clinical‐stage drug products, with no significant impact to biological activity (Bernstein and Qazi 2010). The impact of *O*‐linked mannosylation differs by molecule, but single‐mannose addition has been shown to not induce immunogenic reaction (Cukan et al. 2012), as higher eukaryotes could have similar modifications (Martinez et al. 2007). Nevertheless, further characterization and engineering could be pursued to minimize this posttranslational modification common to yeast. The feasibility of *O*‐linked glycoengineering has been demonstrated (Hamilton et al. 2013), and the homogeneity of resulting *O*‐glycome could be advantageous for biobetter development (Torres‐Obreque et al. 2022).

We have demonstrated the ability to adapt aglycosylated mAbs for high‐quality production in *K. phaffii*. The comparability between yeast‐ and CHO‐secreted products provides additional context to advance alternative hosts for mAb production. The characterizations and engineering presented here establish one approach to producing high‐quality, full‐length mAbs using *K. phaffii*. This intrinsic improvement in the specific productivity of secreted intact mAbs should facilitate new approaches to maximize the space‐time yields with this alternative host to advance rapid development cycles for new biologics and expand applications for global production of low‐cost, high‐quality mAbs.

## Author Contributions

N.C.D., Y.Y., K.R.L., and J.C.L. developed the concepts and designed the study. N.C.D., J.R.B., Y.Y., T.L., R.S.J., and C.M.E. created vectors and yeast strains. E.L. and M.T. created CHO vectors and strains and performed CHO strain cultivation. S.R.A., N.C.D., Y.Y., and M.K.T. performed yeast strain cultivation and protein characterization. Y.Y., N.C.D., and J.C.L. wrote the manuscript.

## Conflicts of Interest

Kerry R. Love is a current employee at Sunflower Therapeutics PBC. J. Christopher Love has interests in Amplifyer Bio, Sunflower Therapeutics PBC, Honeycomb Biotechnologies, OneCyte Biotechnologies, QuantumCyte, and Repligen. J. Christopher Love's interests are reviewed and managed under MIT's policies for potential conflicts of interest.

## Supporting information

Supporting information.

Supporting information.

## Data Availability

The data that support the findings of this study are available from the corresponding author upon reasonable request.
