# Supplementary material for: Adaptation of Aglycosylated Monoclonal Antibodies for Improved Production in Komagataella phaffii
Source: Biotechnol Bioeng. 2024 Nov 14;122(2):361–72. doi: 10.1002/bit.28878 (PMC11718428; doi:10.1002/bit.28878)

**Adaptation of Aglycosylated Monoclonal Antibodies for Improved Production in *Komagataella phaffii***

Supplemental Information

Yuchen Yang^1,2^, Neil C. Dalvie^1,2^, Joseph R. Brady^1,2^, Christopher A. Naranjo^2^, Timothy Lorgeree^2^, Sergio A. Rodriguez-Aponte^2,3^, Ryan S. Johnston^2^, Mary Kate Tracey^2^, Carmen M. Elenberger^2^, Eric Lee^4^, Mark Tié^4^, Kerry R. Love^1,2^, J. Christopher Love^1,2^

^1^Department of Chemical Engineering, Massachusetts Institute of Technology, Cambridge, MA 02139, USA

^2^The Koch Institute for Integrative Cancer Research, Massachusetts Institute of Technology, Cambridge, MA 02139, USA

^3^Department of Biological Engineering, Massachusetts Institute of Technology, Cambridge, MA 02139, USA

^4^Biogen, Cambridge, MA 02142, USA

*Correspondence to: [clove@mit.edu](mailto:clove@mit.edu)

Contents

**Figure S1.** Custom vectors for genomic integration of mAb light chain and heavy chain.

**Figure S2.** N-terminal variants due to incomplete signal peptide cleavage.

**Figure S3.** Mass spectra of trastuzumab heavy chain pre- and post-PNGase treatment.

**Figure S4.** Presence of heavy chain O-linked glycosylation confirmed by enzymatic digestion.

**Figure S5.** Tandem MS mass spectra of an O-glycosylated peptide in trastuzumab heavy chain.

**Figure S6.** Mutations in the O-glycosylated peptide do not eliminate O-glycosylation.

**Figure S7.** Sequence analysis of trastuzumab heavy chain and proteolysis in its Fc domain.

**Figure S8.** Effects of IgG1 light chain and heavy chain copy number.

**Figure S9.** N-terminal variants reduced with amino acid addition.

**Figure S1.** Custom vectors for genomic integration of mAb light chain and heavy chain.


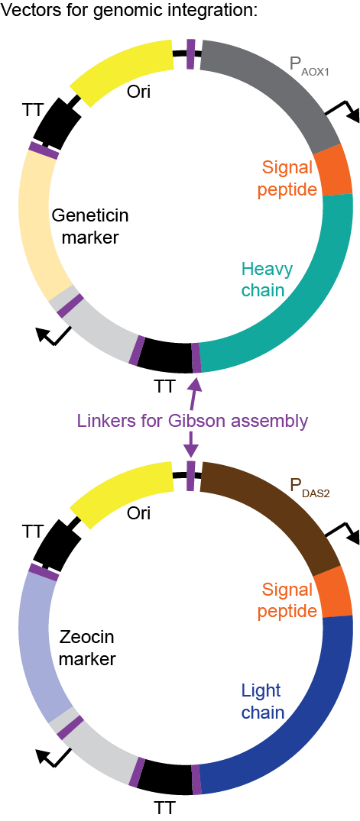


**Figure S2.** N-terminal variants due to incomplete signal peptide cleavage.

preOST1-proαSP was used for both light and heavy chains. N-terminal extensions, as detected by intact LC-MS, are marked in the sequence.


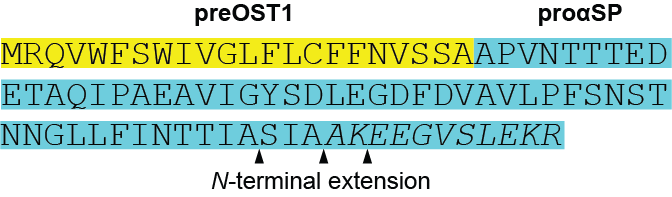


**Figure S3.** Mass spectra of trastuzumab heavy chain pre- and post-PNGase treatment.

PNGase was used to release N-linked glycosylation on trastuzumab heavy chain, revealing its N-terminal variants.


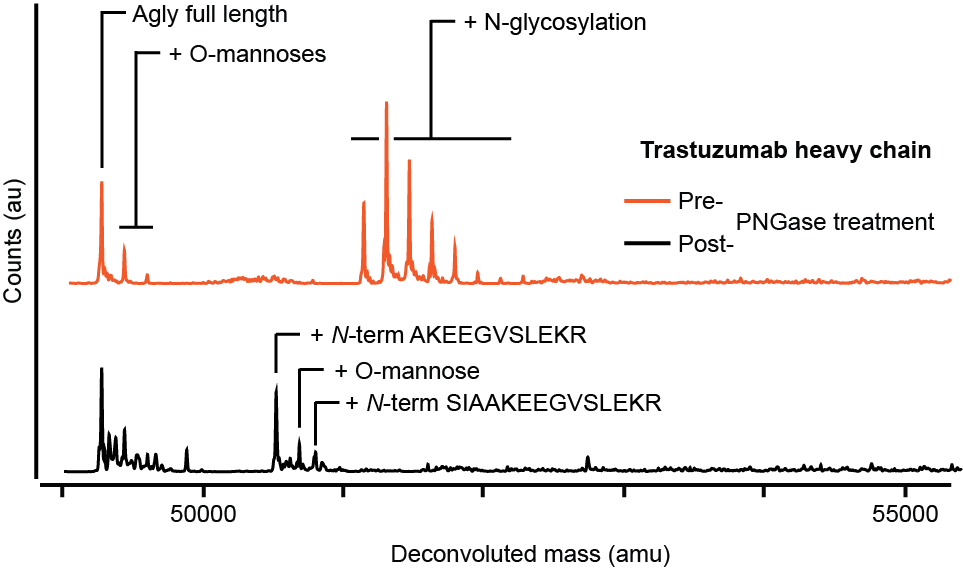


**Figure S4.** Presence of heavy chain O-linked glycosylation confirmed by enzymatic digestion.

Reduced SDS-PAGE of Protein A-purified trastuzumab before and after treatment by PNGase and by wide-acting Jack Bean Mannosidase (JBM, α1-2,3,6-mannosidase). PNGase releases N-linked glycans, while JBM cleaves N- and O-linked mannoses.


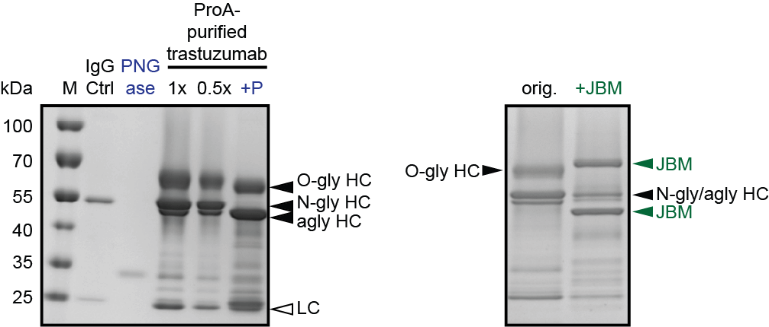


**Figure S5.** Tandem MS mass spectra of an O-glycosylated peptide in trastuzumab heavy chain.

In-gel trypsin digestion of two bands of interest, followed by LC-MS/MS analysis of one peptide of interest for its differential O-linked mannosylation.


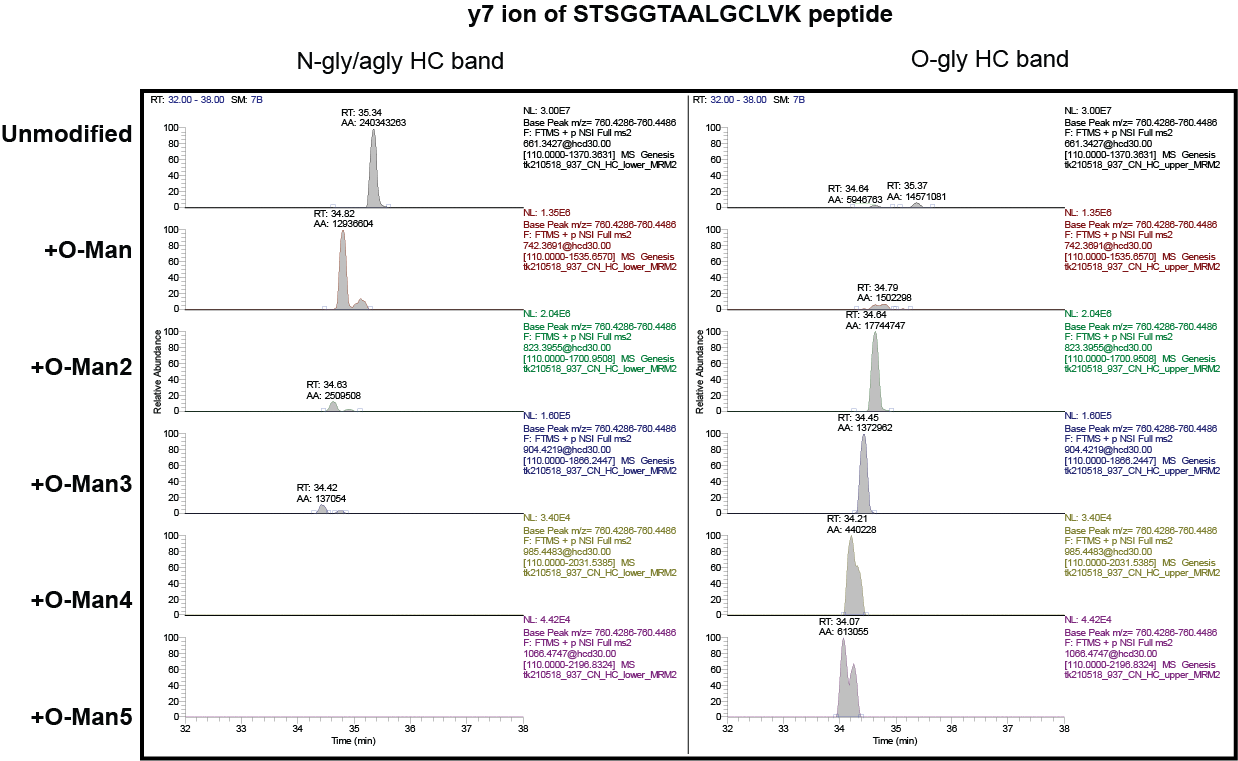


**Figure S6.** Mutations in the O-glycosylated peptide do not eliminate O-glycosylation.

Mutating Ser and/or Thr residues in the peptide of interest had no significant impact on heavy chain O-linked glycosylation.


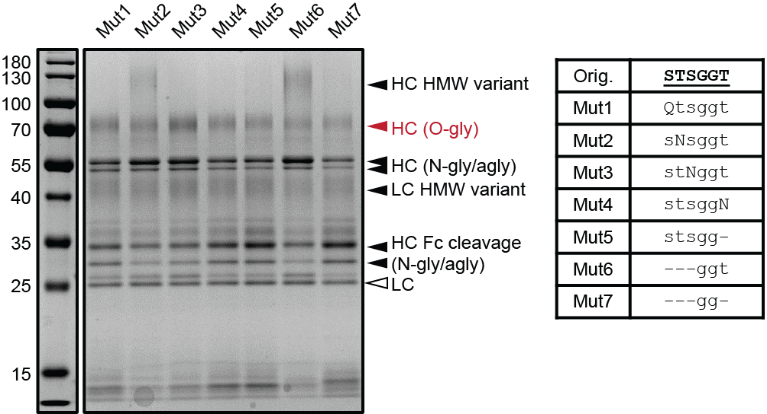


**Figure S7.** Sequence analysis of trastuzumab heavy chain and proteolysis in its Fc domain.

Intact LC-MS mass spectrum of trastuzumab heavy chain cleavage, which occurs at a dibasic Kex2p cleavage site.


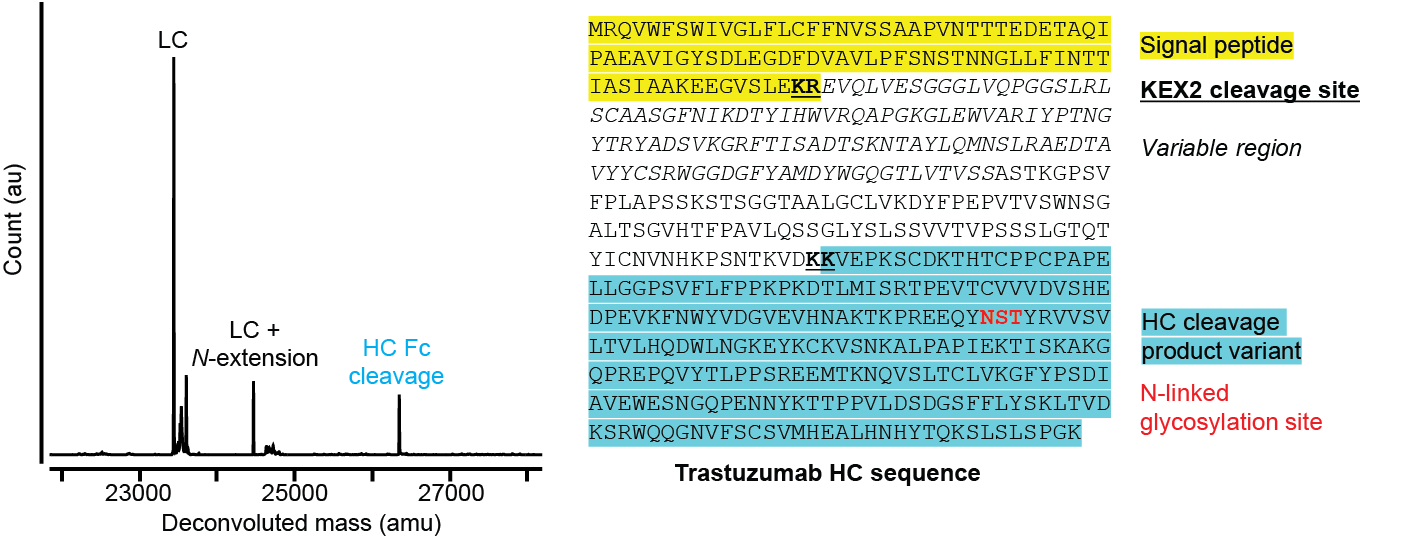


**Figure S8.** Effects of IgG1 light chain and heavy chain copy number.

Reduced SDS-PAGE of culture supernatants of *K. phaffii* clones with different light chain and heavy chain copy number. Copy number was approximated by transformant growth and colony size on antibiotic selection plates, with example colony sizes shown.


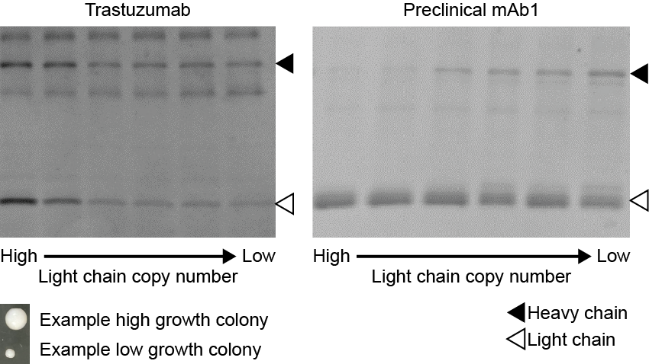


**Figure S9.** N-terminal variants reduced with amino acid addition.

Intact LC-MS mass spectra of Protein A-purified trastuzumab variants secreted from *K. phaffii*. These samples were treated with PNGase after purification to remove all N-linked glycosylation.


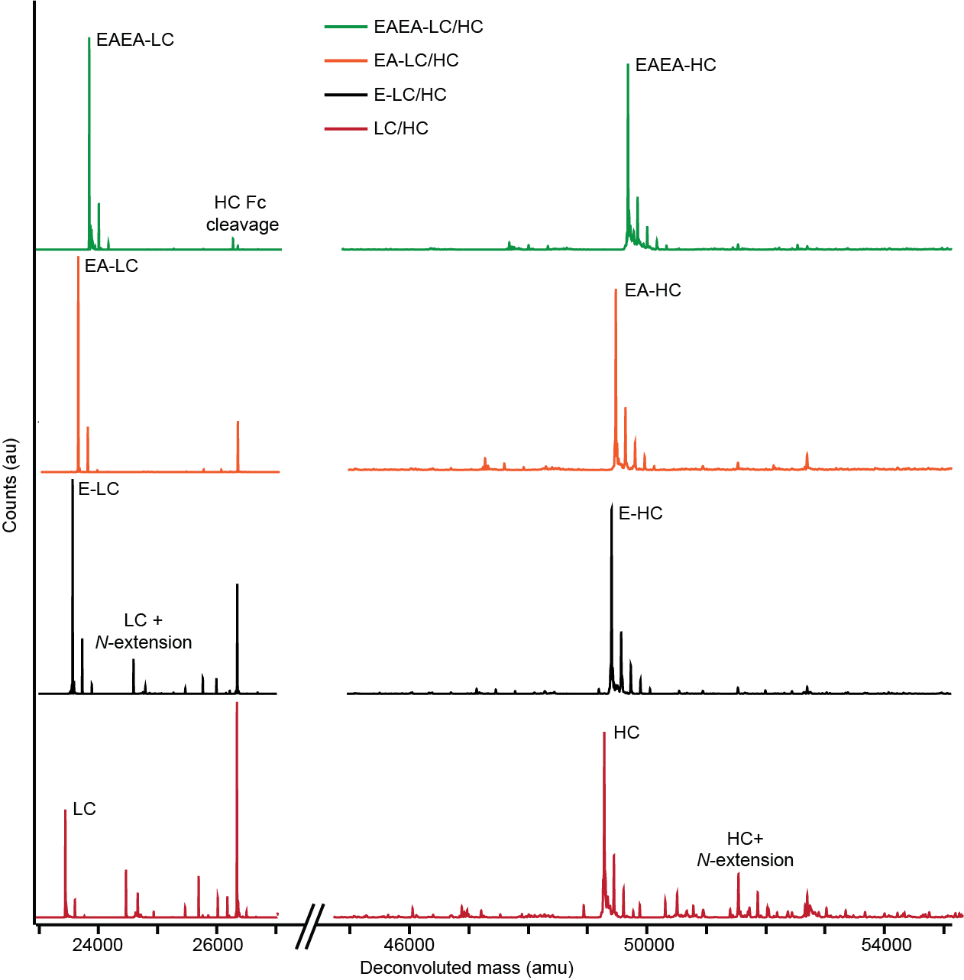

Supplement: Supplementary file 1 — Supporting information. [file BIT-122-361-s001.docx]
