# Supplementary figures and images for: Adaptation of Aglycosylated Monoclonal Antibodies for Improved Production in Komagataella phaffii
Source: Biotechnol Bioeng. 2024 Nov 14;122(2):361–72. doi: 10.1002/bit.28878 (PMC11718428; doi:10.1002/bit.28878)

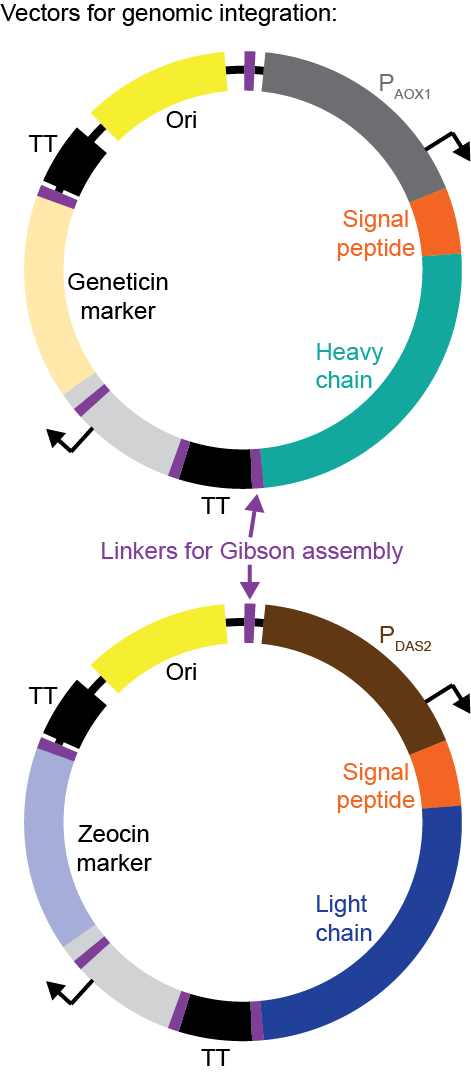

Supplement: Supplementary file 2 — Supporting information. [file BIT-122-361-s002.zip › Supplemental Information/Fig S1.png]

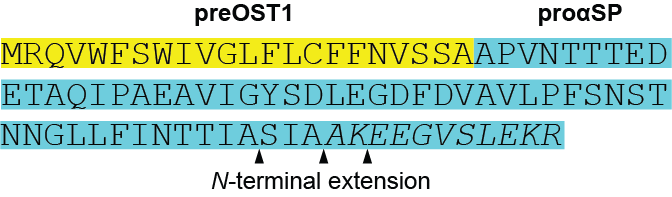

Supplement: Supplementary file 2 — Supporting information. [file BIT-122-361-s002.zip › Supplemental Information/Fig S2.png]

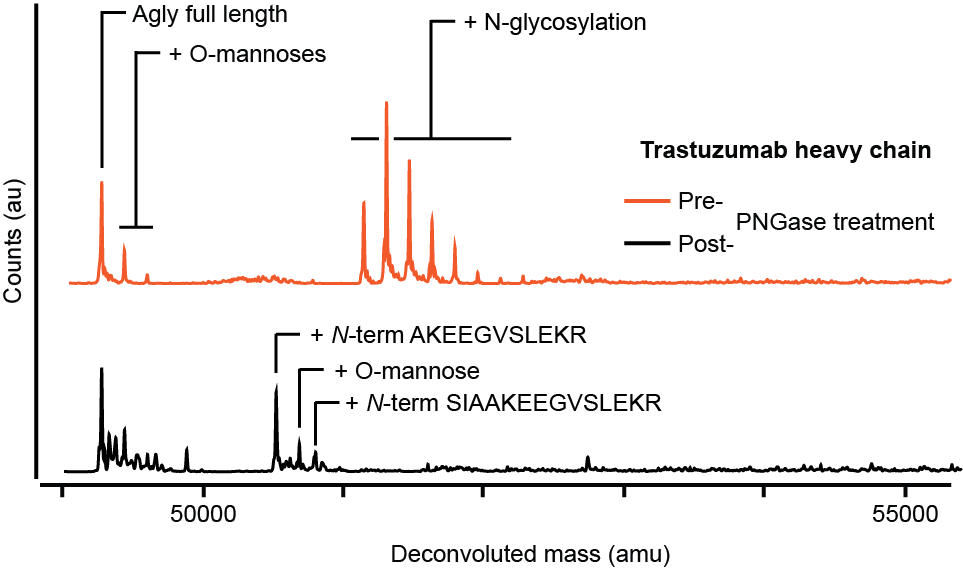

Supplement: Supplementary file 2 — Supporting information. [file BIT-122-361-s002.zip › Supplemental Information/Fig S3.png]

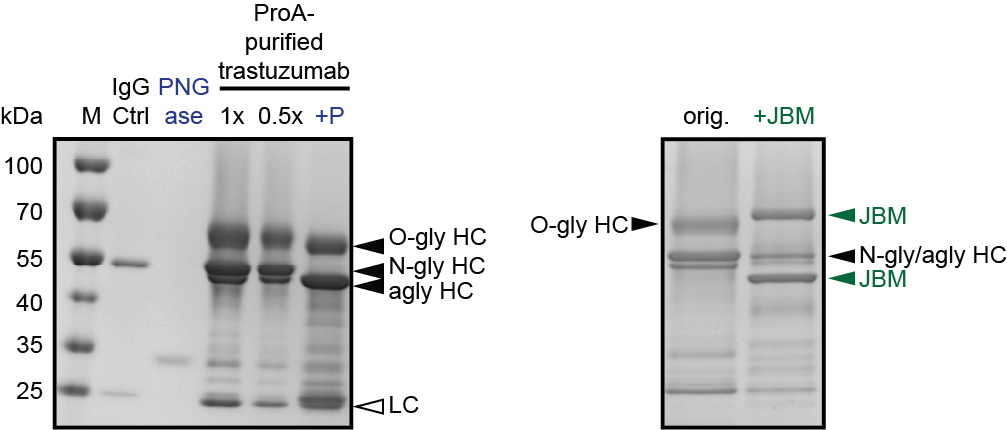

Supplement: Supplementary file 2 — Supporting information. [file BIT-122-361-s002.zip › Supplemental Information/Fig S4.png]

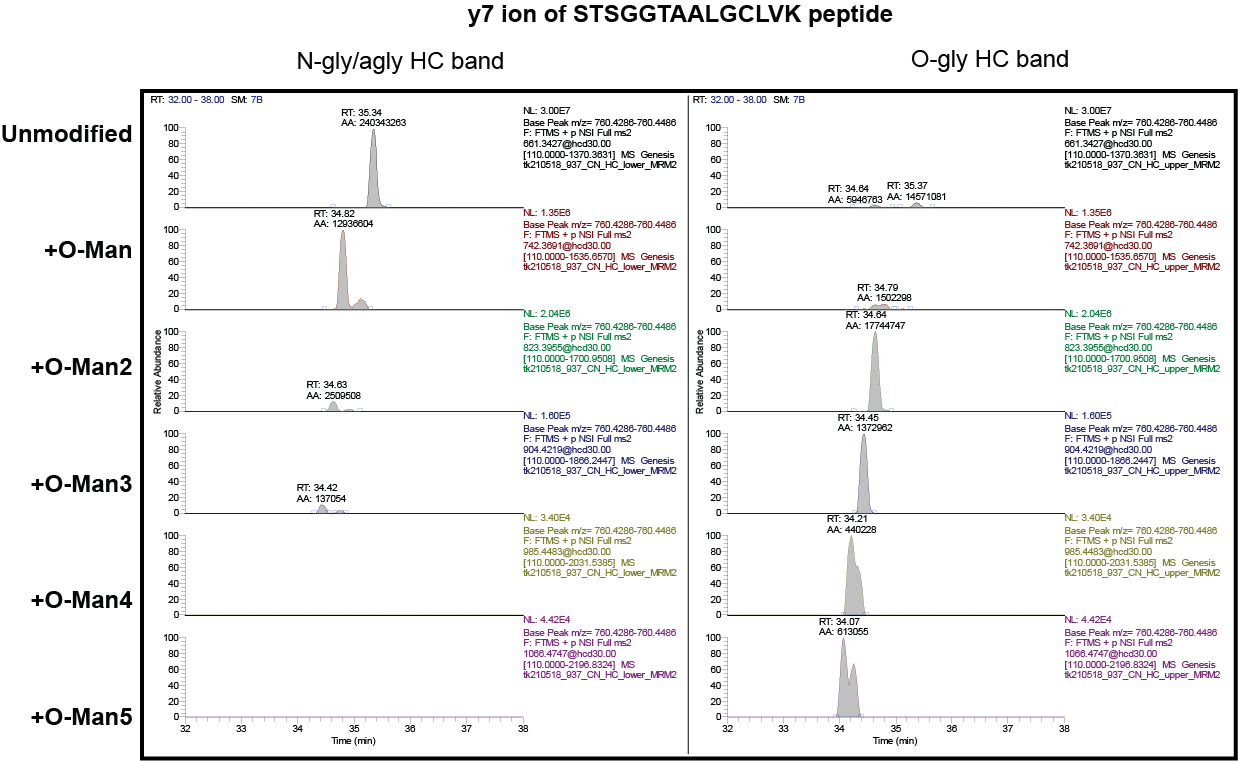

Supplement: Supplementary file 2 — Supporting information. [file BIT-122-361-s002.zip › Supplemental Information/Fig S5.png]

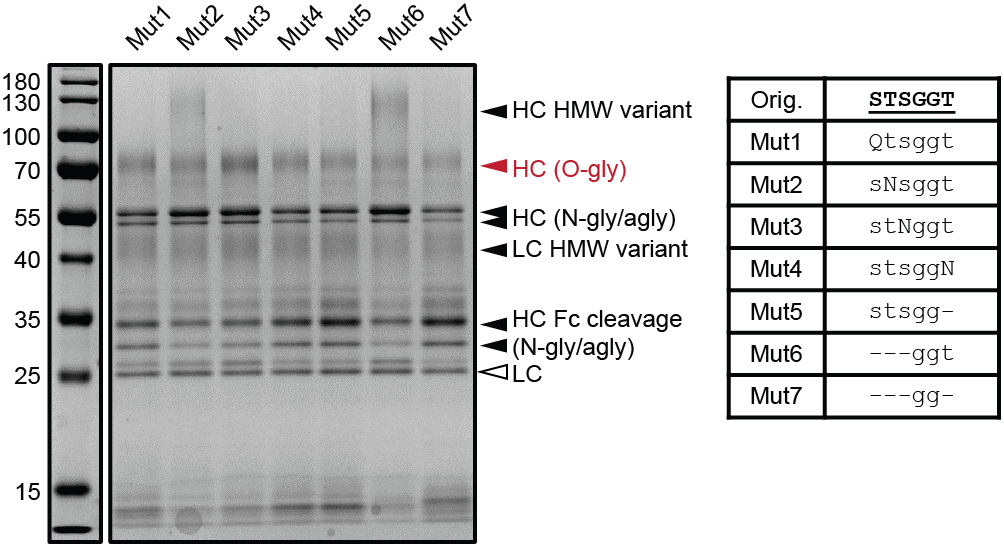

Supplement: Supplementary file 2 — Supporting information. [file BIT-122-361-s002.zip › Supplemental Information/Fig S6.png]

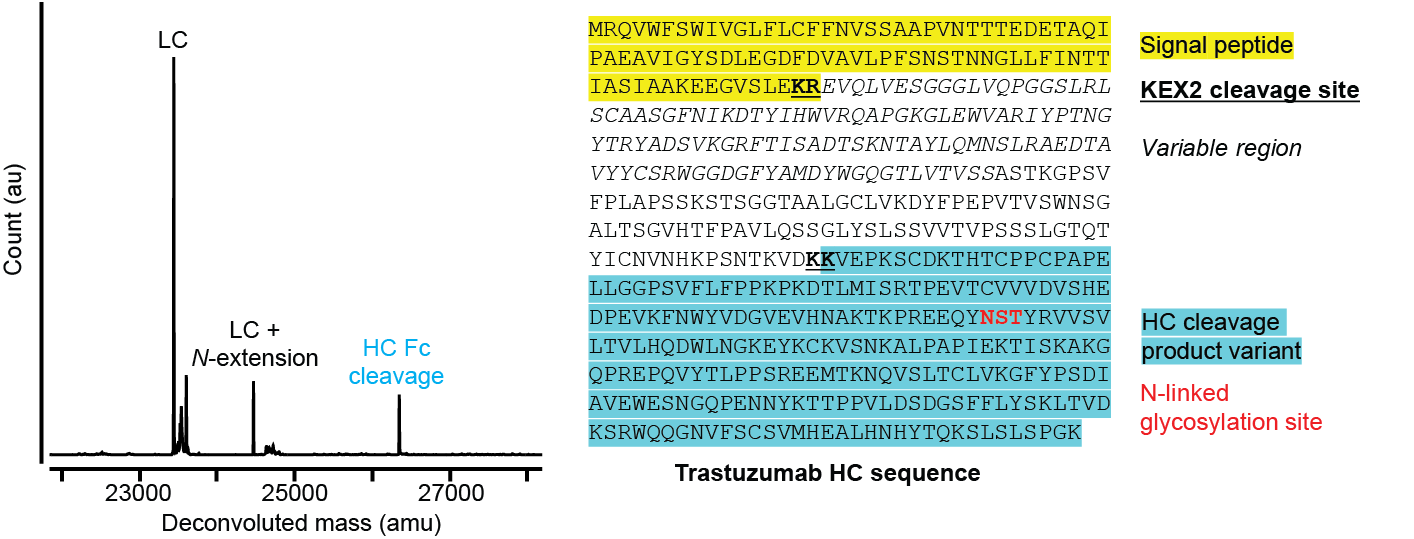

Supplement: Supplementary file 2 — Supporting information. [file BIT-122-361-s002.zip › Supplemental Information/Fig S7.png]

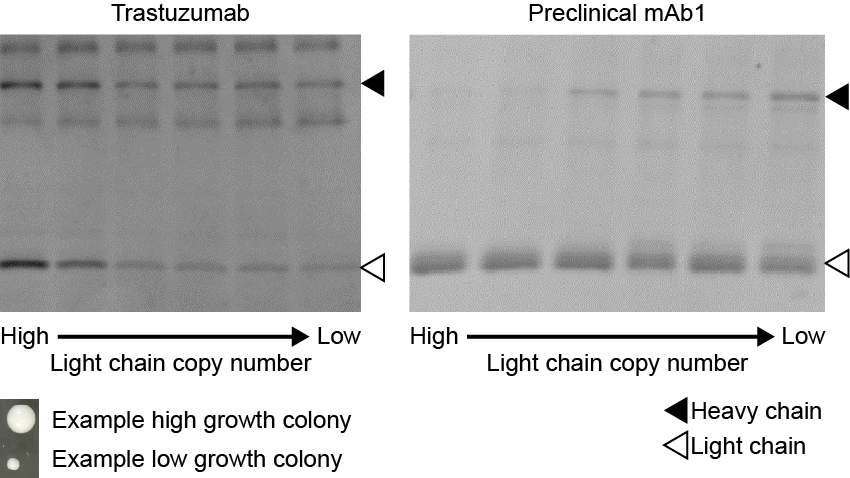

Supplement: Supplementary file 2 — Supporting information. [file BIT-122-361-s002.zip › Supplemental Information/Fig S8.png]

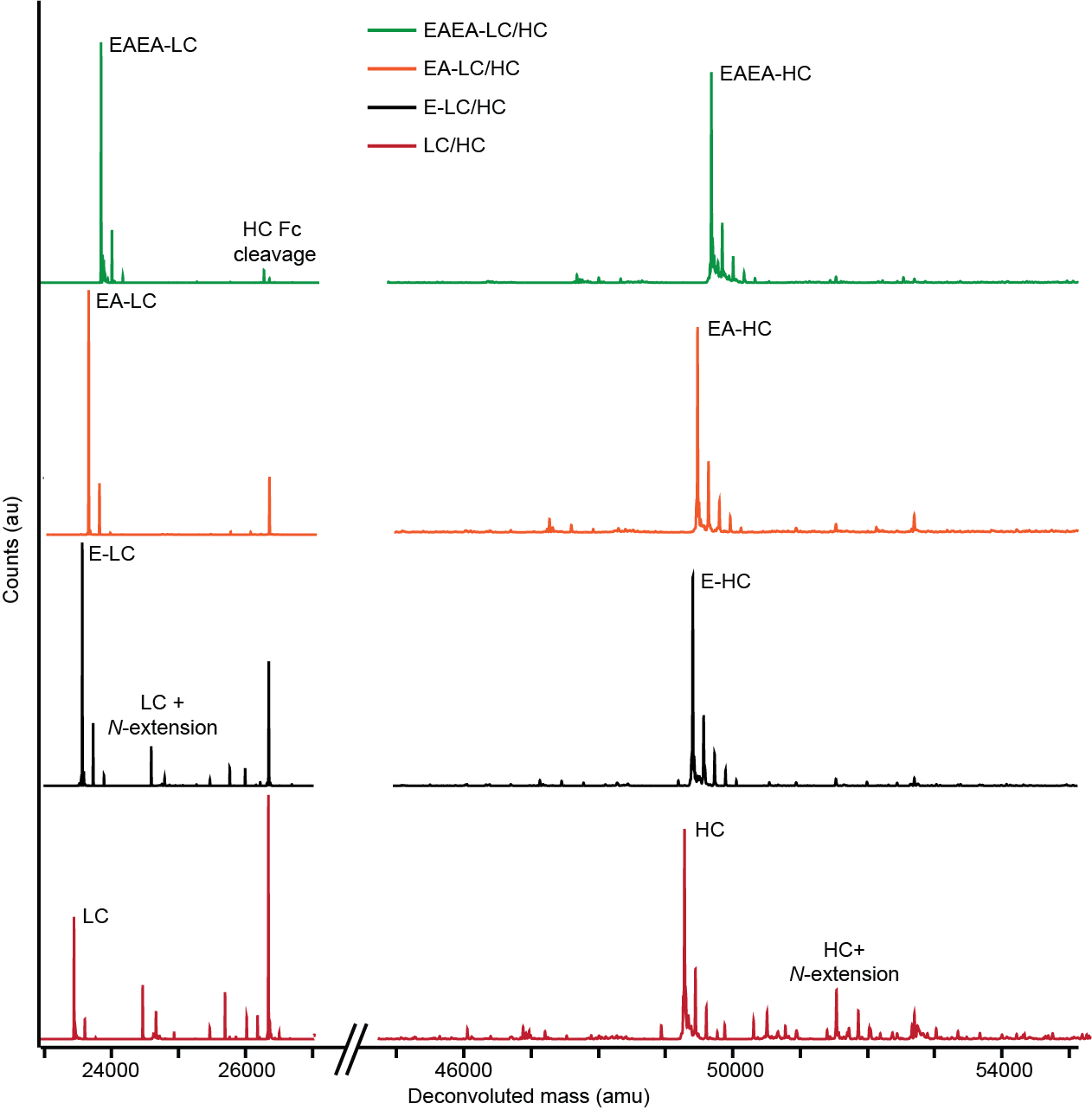

Supplement: Supplementary file 2 — Supporting information. [file BIT-122-361-s002.zip › Supplemental Information/Fig S9.png]
